# Supplementary material for: Resolving the full spectrum of human genome variation using Linked-Reads
Source: Genome Res. 2019 Apr;29(4):635–45. doi: 10.1101/gr.234443.118 (PMC6442396; doi:10.1101/gr.234443.118)
Supplement: Supplemental Material [file supp_gr.234443.118_Supplemental_longranger-2.2.zip › longranger-2.2.tar.bz2!/lr2.2-public/lib/stan/cmdstan/stan/doc/api/html/agrad_2rev_2matrix_2trace__inv__quad__form__ldlt_8hpp.html]

Stan: src/stan/agrad/rev/matrix/trace\_inv\_quad\_form\_ldlt.hpp File Reference


|  |  |
| --- | --- |
|  | Stan  2.5.0  probability, sampling & optimization |


- Main Page
- Related Pages
- Namespaces
- Classes
- Files

- File List
- File Members

All Classes Namespaces Files Functions Variables Typedefs Enumerations Enumerator Friends Macros Pages

- src
- stan
- agrad
- rev
- matrix

Namespaces |
Functions

trace\_inv\_quad\_form\_ldlt.hpp File Reference

`#include <stan/math/matrix/Eigen.hpp>`  
`#include <stan/agrad/rev/var.hpp>`  
`#include <stan/agrad/rev/matrix/LDLT_alloc.hpp>`  
`#include <stan/agrad/rev/matrix/LDLT_factor.hpp>`  
`#include <boost/type_traits/is_same.hpp>`  
`#include <boost/utility/enable_if.hpp>`  
`#include <stan/math/error_handling/matrix/check_multiplicable.hpp>`

Go to the source code of this file.

|  |  |
| --- | --- |
| Namespaces | |
|  | stan |
|  | Probability, optimization and sampling library. |
|  | |
|  | stan::agrad |
|  | Function gradients via reverse-mode automatic differentiation. |
|  | |

|  |  |
| --- | --- |
| Functions | |
| template<typename T2 , int R2, int C2, typename T3 , int R3, int C3> | |
| boost::enable\_if\_c  < boost::is\_same< T2, var >  ::value\|\|boost::is\_same< T3,   var >::value, var >::type | stan::agrad::trace\_inv\_quad\_form\_ldlt (const stan::math::LDLT\_factor< T2, R2, C2 > &A, const Eigen::Matrix< T3, R3, C3 > &B) |
|  | Compute the trace of an inverse quadratic form. More... |
|  | |

## Variable Documentation

trace\_inv\_quad\_form\_ldlt\_impl<T2,R2,C2,T3,R3,C3>\* \_impl

Definition at line 147 of file trace\_inv\_quad\_form\_ldlt.hpp.

stan::math::LDLT\_factor<T2,R2,C2> \_ldlt

Definition at line 80 of file trace\_inv\_quad\_form\_ldlt.hpp.

double \_value

Definition at line 86 of file trace\_inv\_quad\_form\_ldlt.hpp.

Eigen::Matrix<vari\*,R3,C3> \_variB

Definition at line 83 of file trace\_inv\_quad\_form\_ldlt.hpp.

Eigen::Matrix<vari\*,Eigen::Dynamic,Eigen::Dynamic> \_variD

Definition at line 82 of file trace\_inv\_quad\_form\_ldlt.hpp.

Eigen::Matrix<double,R3,C3> AinvB\_

Definition at line 84 of file trace\_inv\_quad\_form\_ldlt.hpp.

Eigen::Matrix<double,C3,C3> C\_

Definition at line 85 of file trace\_inv\_quad\_form\_ldlt.hpp.

Eigen::Matrix<double,Eigen::Dynamic,Eigen::Dynamic> D\_

Definition at line 81 of file trace\_inv\_quad\_form\_ldlt.hpp.

const int Dtype\_

Definition at line 79 of file trace\_inv\_quad\_form\_ldlt.hpp.


---

[ Stan Home Page ]

*© 2011–2014,
Stan Development Team.*
